# Using the PharmCAT tool for Pharmacogenetic clinical decision support

**DOI:** 10.1093/bib/bbad452

**Published:** 2023-12-05

**Authors:** Kevin Tippenhauer, Marwin Philips, Carlo Largiadèr, Murat Sariyar

**Affiliations:** Bern University of Applied Sciences, Quellgasse 21, 2500 Biel, Switzerland; Bern University of Applied Sciences, Quellgasse 21, 2500 Biel, Switzerland; Department of Clinical Chemistry at the Bern University Hospital, Bern, Switzerland; Bern University of Applied Sciences, Quellgasse 21, 2500 Biel, Switzerland

**Keywords:** pharmacogenetics, pharmacogenomics, CPIC guidelines, CDSS, clinical information system

## Abstract

Here, we will provide our insights into the usage of PharmCAT as part of a pharmacogenetic clinical decision support pipeline, which addresses the challenges in mapping clinical dosing guidelines to variants to be extracted from genetic datasets. After a general outline of pharmacogenetics, we describe some features of PharmCAT and how we integrated it into a pharmacogenetic clinical decision support system within a clinical information system. We conclude with promising developments regarding future PharmCAT releases.

Pharmacogenetics is the study of genetic effects on individual drug metabolism and response. Genetic factors’ significance in drug processing varies based on the specific drug, with some having a substantial influence on inter-individual variability in both pharmacokinetics and pharmacodynamics [1]. Notably, approximately 95% of individuals possess genetic variants within known pharmacogenes that hold relevance for drug dosing recommendations [2]. Neglecting these genetic variations during drug prescriptions can lead to severe adverse drug reactions, including potentially fatal ones, or inadequate therapeutic outcomes, such as insufficient pain relief from analgesics. The Clinical Pharmacogenetics Implementation Consortium (CPIC) has assigned preliminary levels for over 500 gene-drug associations and published 26 CPIC guidelines so far, which contain concrete instructions for certain gene-drug combinations [3]. However, the actual implementation of pharmacogenetics in clinics is progressing slowly. The actual problem regarding lacking implementations has shifted from missing genetic knowledge to problems concerning the concrete transfer of this knowledge to the point-of-care [4]. Translation of pharmacogenetic knowledge into the clinical setting is facilitated by pharmacogenetic clinical decision support systems (PGCDSS). One helpful tool in this context is PharmCAT, which generates pharmacogenomics reports in different formats based on Variant Call Format (VCF) files and curated guidelines [5]. PharmCAT addresses the challenges of mapping clinical dosing guidelines to variants to be extracted from genetic datasets [6].

One typical PGCDSS scenario is related to the intervention in the case of prescribing chemotherapy drugs. Say, a patient with colorectal cancer requires treatment with 5-fluorouracil (5-FU), a common chemotherapy drug. However, 5-FU is metabolized by the DPYD enzyme, and genetic variants in the *DPYD* gen can lead to variations in drug metabolism, potentially causing severe toxicity in individuals with specific *DPYD* variants. By using PharmCAT within an PGCDSS following is process is carried out:

1. The patient’s DNA is sequenced (For this purpose, a laboratory order is issued via the laboratory information system) and analyzed using PharmCAT to identify any relevant *DPYD* variants.
2. The PGCDSS integrates the gene-phenotype association from PharmCAT and alerts the oncologist about the patient’s specific *DPYD* genotype and recommends the most appropriate dosage of 5-FU based on the relevant PGx guidelines to minimize the risk of severe side effects.
3. An alternative Treatment is proposed, if a *DPYD* variant significantly impairs metabolism. The PGCDSS suggests alternative chemotherapy options that doesn’t rely on DPYD metabolism, ensuring patient safety and treatment efficacy through tailored decisions.

PharmCAT (https://pharmcat.org) is an open-source tool under the Mozilla Public License (MPL 2.0), mainly developed by the former Pharmacogenomics Research Network Statistical Analysis Resource and the Pharmacogenomics Knowledgebase (PharmGKB). In its current state, it can be run as a single console Java application (jar file) and by invoking one of its modules (Named Allele Matcher, Phenotyper and Reporter). PharmCAT allows the inclusion of external PGx genotype or phenotype data, which needs to be formatted as a tab-delimited value file. Genetic data can be provided by both single-sample and multi-sample VCFs. If the users want to split a multi-sample VCF file, the VCF Preprocessor can separate the file into multiple single-sample VCF files on demand. The knowledge base of the system consists of the allele and phenotype definitions as well as the curated guideline recommendations. As a single console application, the alleles are interpreted, and reports with genotype-based prescribing recommendations are generated (Figure 1). For example, for *CYP2C19* over 30 different alleles are known (see https://www.pharmvar.org/gene/CYP2C19) and it is important to automate interpretation when assessing the variants simultaneously. The current version of PharmCAT and a comprehensive log of all updates can be accessed through the PharmGKB GitHub repository, available at https://github.com/PharmGKB/PharmCAT.

**Figure 1:**
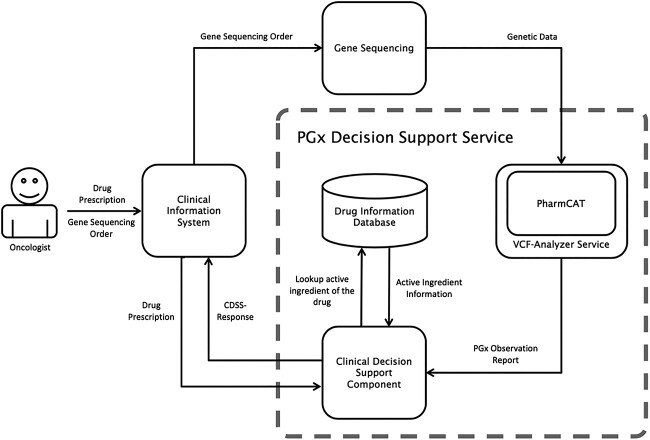
To use PharmCAT within our CIS eco system, the user (Oncologist) places a drug prescription and a gene sequencing order. The CIS then dispatches the sequencing order to the sequencer while directing the prescription order to the Clinical Decision Support System (CDSS) component. As soon as new genetic data becomes available, PharmCAT carries out the annotation process and forwards the resulting pharmacogenetic observation report to the CDSS. The CDSS, in turn, queries the drug information database to retrieve the active ingredient associated with the prescribed drug. If there is a matching phenotype found in the pharmacogenomic observation report for the drug’s active ingredient, the CDSS promptly generates a personalized warning message, which is subsequently relayed to the CIS for further action’.

We have implemented PGCDSS using PharmCAT at the Institute of Clinical Chemistry at the University Hospital in Bern, Switzerland [7]. We decided to focus on chemotherapy drugs, especially on 5-FU, due to the decision of one of the co-authors (see below). Figure 1 depicts the central aspects of our PGCDSS system (the code of our system can be accessed via https://github.com/sym33/PharmCatWrapper_CGM). In order to allow the PharmCAT tool to communicate with the CGM Phoenix clinical information system (CIS) through Health Level Seven International Version 2 (HL7v2) OBR (Observation request) and ORU (Observation Result) messages, a container around PharmCAT was built, enabling it to run as a background service. The necessary extension for such a background service capability was realized by using the Spring Framework together with the HAPI API v2.4 for the HL7 message interface and the Apache Commons Daemon software. To validate our prototype, we have set up a test tool to create patient-specific genetic data and check if the PGCDSS works as expected. Warnings were issued directly to the physician in the case of relevant oncology drug prescriptions within the CIS. One central advantage of a CDSS over the standard PharmCAT reports within a CIS is the ease with which the warnings can be adapted to the needs of the user from a process-oriented perspective. One of the co-authors, Carlo Largiadèr, is a PGx expert at the university hospital Bern and responsible for the content in the PGx recommendations/warnings. He assumed a pivotal role in shaping the content of recommendations and warnings. Given his active engagement with PharmGKB, he oversees updates pertinent to this content. Furthermore, he played a crucial role in the decision-making process concerning the inclusion of chemotherapy drugs, driven by the demand from oncologists seeking support in the realm of PGx-based drug selection.

The configuration files of PharmCAT allowed us to adapt the pharmacogenetic rules to our needs. The JSON reports generated by PharmCAT contain the predicted patient’s phenotype, the affected drugs, recommended actions, and some further meta-data. From these reports, we extracted the metabolic phenotype of the patient (there are more than metabolism genes covered by PharmCAT) and the associated genetic diplotypes/genotypes to provide a succinct and specific warning in the prescription process. The embedded PharmCAT service proved to be an effective solution for extracting relevant phenotypes and genotypes from VCF files and sending them as an ORU message over the laboratory interface into the database of the CIS. PharmCAT is still in active development, and for a stable PGCDSS service, appropriate handling of warnings is necessary. For instance, a trisomy in the VCF file can warning about an uncatalogued single nucleotide variant (SNV). The release cycle is promising in this regard.

Integrating PharmCAT into our PGCDSS showed that this tool is useful for providing context related PGx guideline information (e.g. from CPIC or the Dutch Pharmacogenetics Working Group), but adaptations to other guidelines would require significant efforts. Further, external additions to the tool, such as those mentioned above, are important to ensure effectiveness in a productive environment. As PharmCAT is further developed, it has the potential to be an essential tool for many PGCDS, especially when an HL7/(Fast Healthcare Interoperability Resource (FHIR) output is provided, which seems to be under way [8].

**Key Points**

- PharmCAT is a Java console application for mapping clinical dosing guidelines to gene variants.
- It is important to automate interpretation when assessing the gene variants simultaneously.
- Integrating PharmCAT into a clinical information system still involves some hurdles.
- PharmCAT is in active development and promises to allow an HL7/FHIR output soon.

## Data Availability

The patient-related data used during the current study are not publicly available due to privacy reasons. All other data are available via https://github.com/sym33/PharmCatWrapper_CGM.

## Author Biographies

**Kevin Tippenhauer** is a research associate in biomedical informatics at the Department of Medical Informatics at the University of Applied Sciences, Bern, Switzerland.

**Marwin Philips** is a team leader at CompuGroup Medical AG, responsible for the development of parts of the clinical information systems, Bern, Switzerland.

**Carlo Largiadér** is head of Liquid Biobank Bern, vice director of the Bern Center for Precision Medicine, and professor at the Department of Clinical Chemistry at the Bern University Hospital, Bern, Switzerland.

**Murat Sariyar** is a researcher in bioinformatics and data science & professor at the Department of Medical Informatics at the University of Applied Sciences, Bern, Switzerland.
